# Coexistence of Acute Appendicitis and Sigmoid Diverticulitis

**DOI:** 10.7759/cureus.47642

**Published:** 2023-10-25

**Authors:** Tatiana Fernandez Trokhimtchouk, Álvaro Morillo Cox, Luis F Flores, Daniella Reinoso Brito, Andres Andrade

**Affiliations:** 1 General Surgery, Universidad Internacional del Ecuador, Quito, ECU; 2 General Practice, Universidad Internacional del Ecuador, Quito, ECU; 3 General and Colorectal Surgery, Axxis Hospital, Quito, ECU

**Keywords:** computed tomography, appendectomy, abdominal pain, diverticulitis, appendicitis

## Abstract

In recent years, there has been a notable increase in acute diverticulitis cases, attributed to modern lifestyles and improved diagnostic techniques. We present a rare case of concurrent acute appendicitis and diverticulitis in a 35-year-old male who came to the emergency department with abdominal pain. While appendicitis typically requires surgery, diverticulitis is often managed conservatively. Computed tomography is key in diagnosis and decision-making. Despite their differing treatments, cases like this challenge the perception of their rarity.

This case prompts consideration of multifocal abdominal pathology. Recognizing concurrent appendicitis and diverticulitis is crucial for guiding appropriate diagnostic and treatment strategies, potentially including non-operative management in select cases.

## Introduction

Appendicitis and diverticulitis stand as two of the most prevalent etiologies for acute abdomen frequently encountered in the emergency room setting. While these conditions individually contribute significantly to the burden of acute abdominal pathology, the occurrence of both simultaneously is considered exceedingly rare. Appendicitis requires a surgical approach for resolution, in contrast with the conservative management typically employed for uncomplicated diverticulitis [1,2].

Acute appendicitis, characterized by inflammation of the vermiform appendix, is a well-recognized surgical emergency. Prompt surgical intervention remains the cornerstone of treatment, resulting in favorable outcomes when executed expeditiously [3]. Conversely, uncomplicated diverticulitis, a manifestation of diverticular disease, often entails a more conservative management strategy, which may include antibiotics, dietary modification, and close clinical observation [4]. These divergent treatment modalities underscore the importance of accurate diagnosis and differentiation between the two entities.

Given their disparate management paradigms, the co-occurrence of appendicitis and diverticulitis presents a diagnostic and therapeutic challenge. Limited literature exists addressing this unique clinical scenario, emphasizing the rarity of such cases. Therefore, we present a case of concurrent acute appendicitis and uncomplicated diverticulitis, highlighting the clinical presentation, diagnostic approach, and successful surgical intervention.

## Case presentation

A 35-year-old male, with a history of radical right orchiectomy for seminoma, presented to the emergency department with a 24-hour history of abdominal pain localized in the right and left iliac fossas. The pain had progressively worsened in the last few hours, accompanied by an increase in bowel movement frequency. He denied experiencing any other associated symptoms.

On examination, tenderness was noted in the lower abdomen, along with positive appendicular signs (McBurney’s, Blumberg’s, Valsalva), and rebound tenderness was elicited on palpation of the left iliac fossa.

Blood analyses revealed leukocytosis (13,930/mm^3^, N: 4,587-10,060/mm^3^) with a mild neutrophil predominance (67.1%, N: 38-62%). There was a marked inflammatory response, as indicated by a C-reactive protein level of 100.1 mg/dL (N: <1.0 mg/dL).

**Figure 1:**
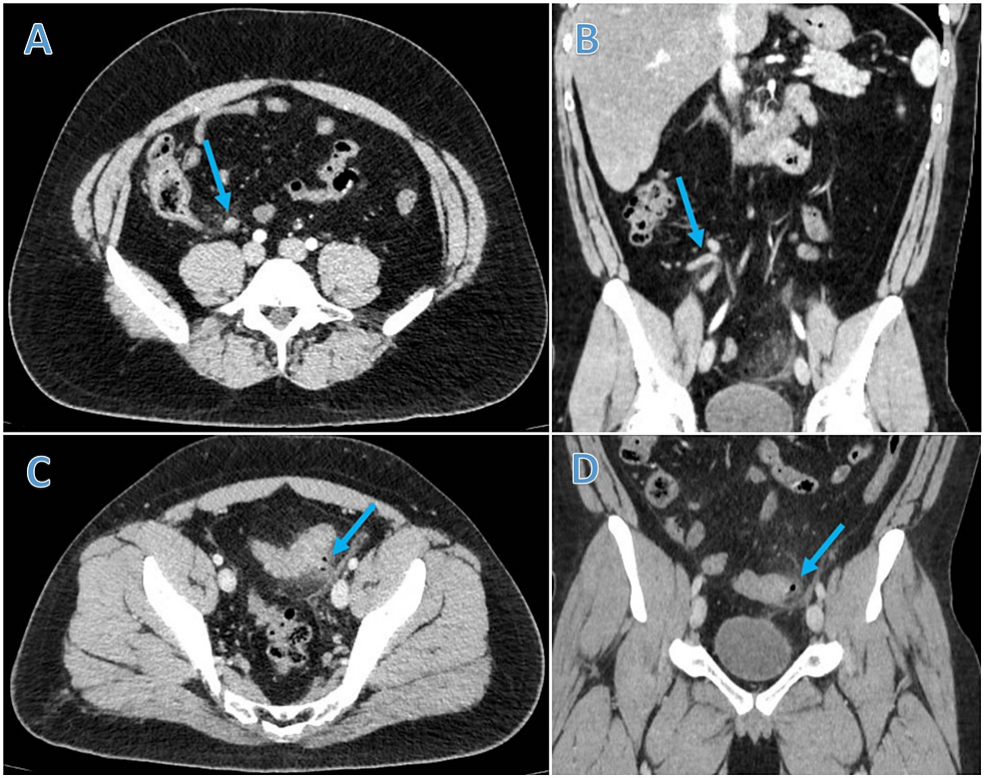
CT scan In Panel A, an axial view during the arterial phase highlights the inflamed appendix, indicated by the arrow. This inflammation is also evident in the coronal view depicted in Panel B. Panels C and D further illustrate the presence of inflamed diverticula, as indicated by the arrow.

A contrast-enhanced computed tomography (CT) of the abdomen and pelvis (Figure 1) demonstrated a retrocecal appendix, measuring 10 mm in diameter, with adjacent mesenteric fat stranding, suggestive of acute appendicitis. Additionally, an area of bowel wall thickening near the descending-sigmoid junction, associated with diverticula and surrounding fat stranding, was noted, consistent with acute uncomplicated diverticulitis (Hinchey IA).

Intravenous fluids were initiated, and the patient was started on antibiotics (ampicillin + sulbactam 3 g IV) and provided with analgesia. A diagnostic laparoscopy was proposed.

**Figure 2:**
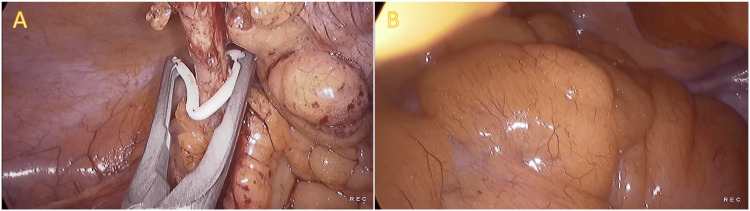
Intraoperative findings Panel A shows the base of the inflamed appendix being ligated. Panel B shows the sigmoid colon covered by small bowel mesentery, which could not be mobilized.

The surgical exploration unveiled an inflamed appendix, as depicted in Figure 2. Furthermore, it was observed that the sigmoid colon was adhered to the lateral wall, ensconced by the mesentery of the small bowel, rendering mobilization unfeasible. Given these findings, no further intervention was undertaken in that area. Subsequently, a laparoscopic appendectomy was executed without encountering any complications.

The patient's postoperative course was uneventful. Liquid oral intake was reintroduced six hours after the surgery and was well-tolerated. The patient was discharged on postoperative day 3, with oral antibiotics prescribed for an additional five days.

The histopathology report confirmed a suppurative acute appendicitis with fibrous obliteration of the tip, along with evidence of acute serositis.

## Discussion

The coexistence of acute appendicitis and uncomplicated diverticulitis in the same patient is a rare clinical entity, often posing a diagnostic challenge. We present a case that exemplifies this unusual occurrence, prompting a comprehensive discussion on its clinical implications and management strategies.

To the best of our knowledge, only a limited number of cases with concurrent appendicitis and diverticulitis have been reported in the literature. Notably, a case described by Hussain et al. in 2016 shares similarities with our present case [5]. In their report, they detail the management of a patient presenting with simultaneous acute appendicitis and diverticulitis, who underwent diagnostic laparoscopy finding acute phlegmonous appendicitis, as well as thickened and inflamed distal descending colon without perforation, the appendectomy was completed and no intervention was performed on the colon. This case, along with our own, underscores the significance of recognizing the potential coexistence of these pathologies, emphasizing the need for a thorough diagnostic workup.

Furthermore, in a review of the literature, we identified another case of concurrent appendicitis and diverticulitis reported by Shademan et al. in 2013, as depicted in an image of a CT in their article [6]. Similarly, this case was successfully managed with antibiotics and appendectomy. This additional case, although not the primary focus of their study, further substantiates the notion that this dual pathology may not be as rare as previously believed.

While surgical intervention remains the cornerstone of treatment for acute appendicitis, there is a growing body of evidence supporting the non-operative management of uncomplicated cases with antibiotics [7]. This approach holds the potential to resolve appendiceal inflammation without necessitating surgical intervention. In instances where both conditions coexist, it is plausible that non-operative management of appendicitis could lead to its spontaneous resolution. This may account for the scarcity of data regarding cases where both pathologies co-occur.

According to Turner et al., there has been a noted rise in the incidence of acute diverticulitis in recent years, potentially attributed to modern lifestyle factors and enhanced diagnostic capabilities, particularly the increased judicious use of imaging techniques [8]. It is conceivable that there may be instances of underdiagnosis regarding the simultaneous presence of the pathologies under discussion. This could be attributed to the prevailing practice where young male patients suspected of appendicular disease often proceed directly to surgery, bypassing a thorough evaluation with computed tomography.

## Conclusions

The coexistence of acute appendicitis and diverticulitis, though traditionally deemed rare, may be more prevalent than initially perceived. This case underscores the importance of thorough diagnostic evaluation when confronted with atypical presentations of abdominal pain. It challenges the conventional approach of immediate surgical intervention in young male patients with suspected appendicular disease, advocating for a more comprehensive evaluation, including computed tomography.

This case serves as a noteworthy contribution to the existing body of literature, providing clinicians with valuable insights into the management of these uncommon dual pathologies. Additionally, it underscores the significance of considering multifocal abdominal pathology when confronted with atypical clinical presentations. The successful surgical management in this case aligns with the standard of care for acute appendicitis. However, the growing body of evidence supporting non-operative management for uncomplicated cases raises intriguing prospects for future exploration, especially in cases with concurrent pathologies.

## References

[1] Di Saverio S, Podda M, De Simone B (2020). Diagnosis and treatment of acute appendicitis: 2020 update of the WSES Jerusalem guidelines. World J Emerg Surg.

[2] Hall J, Hardiman K, Lee S (2020). The American Society of Colon and Rectal Surgeons clinical practice guidelines for the treatment of left-sided colonic diverticulitis. Dis Colon Rectum.

[3] Andersson RE (2007). The natural history and traditional management of appendicitis revisited: spontaneous resolution and predominance of prehospital perforations imply that a correct diagnosis is more important than an early diagnosis. World J Surg.

[4] Stollman N, Raskin JB (2004). Diverticular disease of the colon. Lancet.

[5] Hussain AS, Gunter OL, Miller RS, Bonatti HJR (2016). Concurrent acute appendicitis and recurrent acute diverticulitis: a diagnostic challenge. CRSLS Journal of the Society of Laparoscopic and Robotic Surgeons.

[6] Shademan A, Tappouni RF (2013). Pitfalls in CT diagnosis of appendicitis: pictorial essay. J Med Imaging Radiat Oncol.

[7] de Almeida Leite RM, Seo DJ, Gomez-Eslava B (2022). Nonoperative vs operative management of uncomplicated acute appendicitis: a systematic review and meta-analysis. JAMA Surg.

[8] Turner GA, O'Grady MJ, Purcell RV, Frizelle FA (2022). Acute diverticulitis in young patients: a review of the changing epidemiology and etiology. Dig Dis Sci.

